# A Case of Spontaneously Improving Secondary Hemophagocytic Lymphohistiocytosis in an Adult Associated with T-Cell Histiocyte-Rich Large B-Cell Lymphoma

**DOI:** 10.1155/2018/8069182

**Published:** 2018-02-22

**Authors:** Cyrus Askin, Ashley Burris, Clifton Layman, Brian Haney, Jordan Hall

**Affiliations:** ^1^Department of Internal Medicine, San Antonio Military Medical Center, San Antonio, TX, USA; ^2^Department of Pathology, San Antonio Military Medical Center, San Antonio, TX, USA; ^3^Department of Hematology and Oncology, San Antonio Military Medical Center, San Antonio, TX, USA

## Abstract

Secondary hemophagocytic lymphohistiocytosis (HLH) in adults is a rare, often fatal syndrome characterized by widespread immune dysregulation. It is seen as a complication of infections, autoimmune diseases, and malignancies. Among the malignancy-related causes, aggressive T-cell or NK-cell neoplasms are most notable, while B-cell lymphomas are less commonly implicated. We present the case of a 32-year-old male transferred to our facility with concern for HLH. During the first week of his hospitalization, his diagnosis was confirmed and the patient demonstrated spontaneous improvement in his symptoms prompting us to delay therapy while searching for a primary cause. In the second week, the patient deteriorated, leading us to initiate steroid monotherapy in the absence of a cause for his HLH. Meanwhile, pathology results from an excisional lymph node biopsy confirmed a diagnosis of T-cell/histiocyte-rich large B-cell lymphoma (TCHRLBCL). Subsequently, we initiated therapy with dose-adjusted R-EPOCH. The patient achieved a complete remission of both HLH and TCHRLBCL as well as a complete return to his prior functional status. In our review of the literature, this represents only the second documented case of HLH associated with TCHRLBCL and the only documented case of an adult experiencing significant spontaneous recovery in this context.

## 1. Introduction

Hemophagocytic lymphohistiocytosis (HLH) is a rare, hyperinflammatory syndrome characterized by cytopenias with fevers, chills, malaise, myalgias, and diaphoresis with high morbidity and mortality [1]. It can occur as a primary disorder (i.e., familial or genetic HLH) or be secondary to another disease process (i.e., reactive or acquired HLH) [2]. In adults, almost invariably, HLH is acquired in the setting of an infectious, rheumatologic, or malignant process. Although swift identification and therapy are key, the recognition of HLH presents a diagnostic dilemma due to protean presentations mimicking septic shock or systemic manifestations of other diseases [3]. For the above reasons, clinicians must maintain a high index of suspicion in order to make the diagnosis early in its course. However, once HLH is presumed or confirmed, prompt and aggressive therapy with steroids and chemotherapy is generally recommended along with advanced supportive care in many cases.

We present a patient who defied the conventional wisdom as it pertains to HLH. Our patient, despite clearly manifesting HLH based upon laboratory values, complained of a flu-like syndrome without hemodynamic instability or a need for invasive supportive therapies. Furthermore, he began to improve without the initiation of therapy. Even in the face of compelling laboratory data, we embarked on an exhaustive search for an etiology of the patient's HLH prior to initiating therapy, after discussion with experts in the field. Although we ultimately started the patient on steroid monotherapy due to clinical deterioration, our approach gave us the necessary time to find the cause of the patient's HLH and to begin targeted treatment towards both his HLH and the underlying etiology, TCHRLBL [4].

## 2. Case Presentation

A 32-year-old National Guard member stationed in Nevada, with no significant past medical history presented with three weeks of progressive dyspnea, fevers, fatigue, diaphoresis, and weight loss. He was initially treated supportively on an ambulatory basis; however, his symptoms progressed to include high-grade fevers, worsening diaphoresis, and fatigue. Preliminary laboratory evaluation revealed thrombocytopenia (52 × 10^3^/*μ*L), anemia (Hgb of 8.0 g/dL), and marked hyperferritinemia (>8000 ng/dL). Imaging studies were significant for hepatosplenomegaly and diffuse lymphadenopathy. He was subsequently transferred to our tertiary care facility.

We conducted a complete evaluation with suspicion for HLH, including workup for a primary etiology. The patient denied exposure to toxins and fungi and any significant travel history aside from recent duty in Nevada and subsequent travel to our hospital in Texas. He endorsed arthralgias and myalgias as well as subjective fevers and malaise, but was otherwise without complaint. His physical examination was significant for a fever of 101.0°F, jaundice, and scleral icterus without visible rashes or palpable adenopathy. Laboratory findings were significant for white blood cell count of 1.1 × 10^3^/*μ*L (with an absolute neutrophil count of 800 × 10^3^/*μ*L), hemoglobin and hematocrit of 8.5 g/dL and 26.3%, respectively, platelets of 64 × 10^3^/*μ*L, ferritin of >8000 ng/dL, triglycerides of 237 mg/dL, fibrinogen of 111 mg/dL, and a soluble interleukin-2 receptor (sIL-2R) level of 23,670 U/mL. Genetic screening for primary HLH was performed which was negative.

A complete metabolic panel was notable for aspartate aminotransferase of 552 U/L, alanine aminotransferase of 679 U/L, alkaline phosphatase of 798 U/L, total bilirubin of 6.32 mg/dL (direct 5.73 mg/dL), and gamma glutamyl transferase of 230 U/L. Renal function was within normal limits. An infectious workup to include fecal leukocytes, stool culture with ova and parasites, multiple blood cultures, urine culture, fungal cultures (screening for histoplasmosis, coccidioidomycosis, and blastomycosis), *Clostridium difficle* PCR, human immunodeficiency virus antibody, acute and chronic hepatitis panels, Epstein-Barr virus, cytomegalovirus, and parvovirus-B19 testing were all negative. Rheumatologic screening labs including anti-nuclear antibody, erythrocyte sedimentation rate, and C-reactive protein were all within normal limits. A drug screen was also unremarkable.

At initial evaluation, the patient met 7 of 8 criteria for establishing a diagnosis of HLH with a minimum of 5 criteria being required (Table 1). We considered initiating standard treatment for HLH based on the fulfillment of these criteria; however, after arrival to our facility, the patient showed significant, spontaneous improvement. He endorsed subjective improvement in his general symptoms, was afebrile for several days, and had improving laboratory values—specifically near-resolution of his hyperferritinemia and pancytopenia. Given this unexpected change in his clinical course, we reached out to experts in the field who agreed with our plan to defer induction therapy while continuing our evaluation.

A bone marrow biopsy revealed hemophagocytosis without evidence of malignancy. A core biopsy of a left supraclavicular lymph node demonstrated an apparent diffuse proliferation of T-cells and histiocytes with singly distributed, large atypical B-cells. However, since a definitive diagnosis could not be rendered from this limited sample, an excisional biopsy was requested. Meanwhile, he deteriorated (recurrent fevers, night sweats, anemia, neutropenia, increasing fatigue, and a pruritic, morbiliform rash). A biopsy of the rash revealed mild perivascular, periadenexal, and dermal mixed inflammation. Medications that had been started for neutropenic precautions, including trimethoprim-sulfamethoxazole, were held with concern for drug reaction; however, his rashes relapsed and remitted even after cessation of these medications. In the setting of his clinical deterioration, we initiated therapy with high-dose corticosteroids while awaiting final pathologic diagnosis of the excisional biopsy. The patient subjectively and objectively responded to steroid therapy.

**Figure 1:**
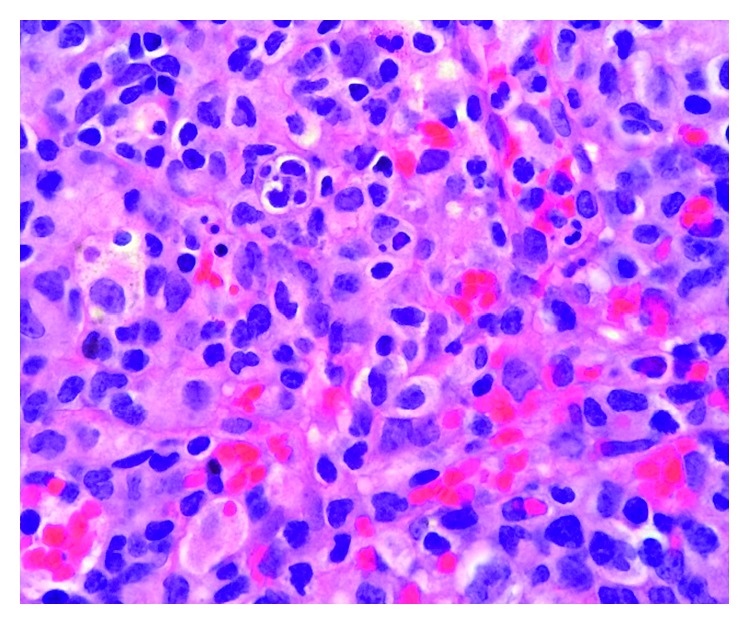
H&E of left supraclavicular lymph node at 100x demonstrating hemophagocytosis with apoptotic debris.

**Figure 2:**
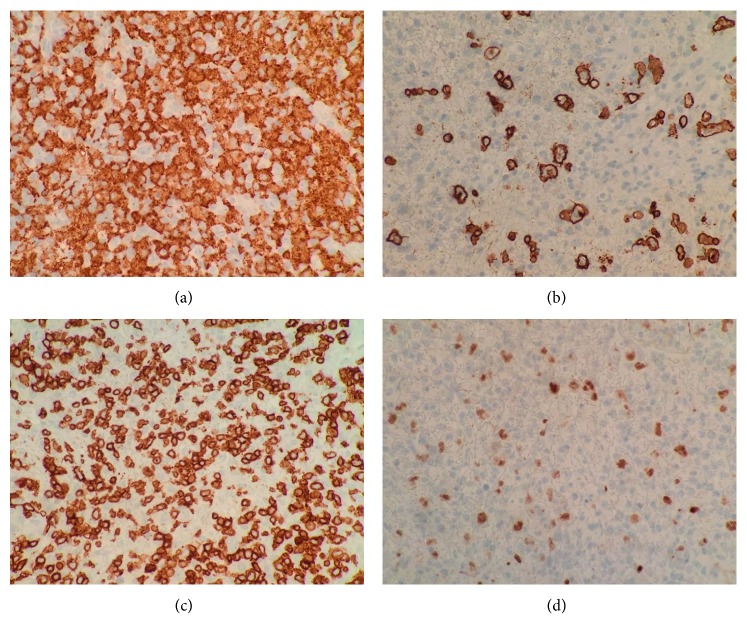
(a) CD3 stain at 40x highlighting the T-cells. (b) PAX5 stain at 40x highlighting the B-lymphocytes. (c) CD20 stain at 40x highlighting the background large B-cells. (d) CD163 stain at 40x highlighting the histiocytes.

On day three of high-dose corticosteroids, pathology results including immunohistochemical evaluation of the excision revealed a completely effaced lymph node with a population of singly distributed large, atypical B-cells expressing CD20, PAX5, BCL6, CD45, and CD15 with CD3+ T-cells and CD163+ histiocytes making up the background cellularity (Figures 1 and 2). The neoplastic B-cells were negative for CD30, CD10, ALK1, and CD138 as well as Epstein-Barr virus encoded RNA (EBER) by chromogenic in situ hybridization. B-cell receptor gene rearrangement studies were positive for a clonal *IGH* gene rearrangement. These findings established a diagnosis of T-cell/histiocyte-rich large B-cell lymphoma with associated HLH. As a result, we initiated chemotherapy with dose-adjusted R-EPOCH (rituximab, etoposide, prednisone, vincristine, cyclophosphamide, and doxorubicin) for comprehensive treatment of both HLH and TCHRLBL. Meanwhile, pathology review was obtained from the National Institutes of Health, confirming the diagnosis. The patient clinically improved during cycle 1 of therapy and was discharged. He returned to his home in Georgia to complete his treatment course where he achieved a complete response of both HLH and TCHRLBL.

## 3. Discussion

As stated earlier, the diagnosis of HLH can be a difficult one to make. Given the myriad of symptoms and laboratory findings associated with this condition, the HLH-94 clinical trial developed certain diagnostic criteria necessary to make the diagnosis of HLH which were revised via HLH-2004 (Table 1) [5]. These criteria are used in the diagnosis of both primary and secondary HLH. In primary or familial HLH, the typical presentation involves an infant, often with a viral prodrome, who has persistently high fevers beyond what would be expected for a viral illness, coupled with a need for aggressive supportive care. If this persists for several days in the setting of a markedly elevated ferritin, the possibility of HLH rises to the forefront and genetic testing is often performed. Individuals with familial HLH generally have one (or more) of several mutations effecting immunomodulation [6]. Some of these include mutations to PRF1, MUNC13-4, RAB27A, STX11, and STXBP2. Specifically, these mutations effect perforin function, the regulation of lytic granules, and vesicular trafficking. Ultimately, treatment of such patients may require hematopoietic stem cell transplant in the absence of recovery with supportive care. However, results have been mixed given the incidence of graft-versus-host disease in infants requiring transplant [7]. Patients with HLH have been documented to have mortalities in excess of 40% even with therapy and aggressive supportive care, worse in the setting of extreme hyperferritinemia (>10,000 ng/dL) [8].

Adult cases of HLH are generally due to a secondary cause such as a rheumatologic condition, infection, or malignancy. It is occasionally confused with adult-onset Still's disease owing to a similar symptomatology and similar “cytokine storm” phenomenon; however, differences in certain lab values and the absence of bone marrow findings distinguish these two conditions [9]. When the constellation of laboratory findings and symptoms characteristic of HLH are secondary to a rheumatologic condition (most often systemic juvenile idiopathic arthritis), it is deemed macrophage activation syndrome (MAS) [10]. This is an important distinction in that these cases are typically best treated by treating the underlying rheumatologic illness which usually entails increasing immunosuppressive therapies and potentially intravenous immunoglobulin (IVIG).

Infections such as Epstein-Barr virus, cytomegalovirus, herpes simplex virus, varicella zoster virus, and human herpes virus 8 are among the more common causes of secondary HLH that have been documented via case reports, making up an estimated 25–30% of these cases [11]. Although less common than viral causes, mycobacterium, bacteria, fungi, and parasites have also been shown to cause secondary HLH and should not be overlooked as potential etiologies [12]. When an infectious cause for HLH is identified, treatment of the illness with antimicrobials may result in resolution of the patient's HLH. If symptoms persist, chemotherapy is recommended.

In a 2014 study by Parikh et al., more than 50% of secondary HLH cases were ascribed to malignancy [13]. Amongst such cases, non-Hodgkin's lymphomas are most commonly implicated—specifically anaplastic large cell lymphoma and natural killer cell lymphomas. A minority of malignancy-related HLH cases are associated with B-cell lymphomas. Of course, cases of HLH associated with malignancy are most effectively treated by way of targeted therapy towards the associated cancer.

Based upon the commonly cited article by Jordan et al. entitled How I Treat Hemophagocytic Lymphohistiocytosis, “prompt initiation of immunochemotherapy is essential to survival [5].” Usual therapy includes dexamethasone with etoposide and might additionally include cyclosporine and intrathecal methotrexate if the patient has evidence of central nervous system involvement. Newer approaches to therapy include using antithymocyte globulin or alemtuzumab; however, these have not become standard options and are more often used in refractory cases [14, 15].

In our review of the English literature, this represents only the second documented case of HLH associated with T-cell/histiocyte-rich large B-cell lymphoma and the only documented case of an adult experiencing significant spontaneous recovery in this context [8]. While steroid monotherapy has been recommended in some instances for HLH therapy, we are not aware of another case where a patient had such markedly abnormal labs yet was clinically stable enough to warrant this approach to treatment. Although a minor aspect of this case, we hope to illustrate the importance of excisional lymph node biopsies in such patients as inadequate tissue sampling can lead to poor outcomes given the significance of this diagnosis. Overall, we believe the complexity and difficulties encountered in diagnosing and treating this patient make his presentation both uniquely interesting and informative for future cases.

## Figures and Tables

**Table 1:** Diagnostic criteria for HLH^∗^.

Fever ≥38.5°C
Splenomegaly
Peripheral blood cytopenia, with at least two of the following:
Hemoglobin <9 g/dL (hemoglobin <10 g/dL for infants <4 weeks)
Thrombocyte count <100,000/*μ*L
Absolute neutrophil count (ANC) <1000/*μ*L
Fasting triglycerides >265 mg/dL and/or fibrinogen <150 mg/dL
Hemophagocytosis in bone marrow, spleen, lymph node, or liver
Low or absent NK-cell activity
Ferritin >500 ng/mL
Elevated soluble CD25 (soluble IL-2 receptor alpha) two standard deviations above age-adjusted laboratory-specific norms (or >2400 U/ml, often cited in the literature)

^∗^≥5/8 criteria must be met for the diagnosis. Adapted from Jordan et al. [5].

## References

[1] Schram A. M., Berliner N. (2015). How I treat hemophagocytic lymphohistiocytosis in the adult patient. *Blood*.

[2] Zhang L., Zhou J., Sokol L. (2014). Hereditary and acquired hemophagocytic lymphohistiocytosis. *Cancer Control*.

[3] Raschke R. A., Garcia-orr R. (2011). Hemophagocytic lymphohistiocytosis: a potentially underrecognized association with systemic inflammatory response syndrome, severe sepsis, and septic shock in adults. *Chest*.

[4] International Agency for Research on Cancer, World Health Organization (2008). *WHO Classification of Tumours of Haematopoietic and Lymphoid Tissues*.

[5] Jordan M. B., Allen C. E., Weitzman S., Filipovich A. H., Mcclain K. L. (2011). How I treat hemophagocytic lymphohistiocytosis. *Blood*.

[6] Filipovich A. H., Chandrakasan S. (2015). Pathogenesis of hemophagocytic lymphohistiocytosis. *Hematology/Oncology Clinics of North America*.

[7] Jordan M. B., Filipovich A. H. (2008). Hematopoietic cell transplantation for hemophagocytic lymphohistiocytosis: a journey of a thousand miles begins with a single (big) step. *Bone Marrow Transplant*.

[8] Devitt K., Cerny J., Switzer B. (2014). Hemophagocytic lymphohistiocytosis secondary to T-cell/histiocyte-rich large B-cell lymphoma. *Leukemia Research Reports*.

[9] Efthimiou P., Paik P. K., Bielory L. (2006). Diagnosis and management of adult onset Still’s disease. *Annals of the Rheumatic Diseases*.

[10] Grom A. A., Mellins E. D. (2010). Macrophage activation syndrome: advances towards understanding pathogenesis. *Current Opinion in Rheumatology*.

[11] Campo M., Berliner N. (2015). Hemophagocytic lymphohistiocytosis in adults. *Hematology/Oncology Clinics of North America*.

[12] Tseng Y. T., Sheng W. H., Lin B. H. (2011). Causes, clinical symptoms, and outcomes of infectious diseases associated with hemophagocytic lymphohistiocytosis in Taiwanese adults. *Journal of Microbiology, Immunology and Infection*.

[13] Parikh S. A., Kapoor P., Letendre L., Kumar S., Wolanskyj A. P. (2014). Prognostic factors and outcomes of adults with hemophagocytic lymphohistiocytosis. *Mayo Clinic Proceedings*.

[14] Ouachée-Chardin M., Elie C., De Saint Basile G. (2006). Hematopoietic stem cell transplantation in hemophagocytic lymphohistiocytosis: a single-center report of 48 patients. *Pediatrics*.

[15] Marsh R. A., Allen C. E., Mcclain K. L. (2013). Salvage therapy of refractory hemophagocytic lymphohistiocytosis with alemtuzumab. *Pediatric Blood & Cancer*.

